# STAT1 inhibits human hepatocellular carcinoma cell growth through induction of p53 and Fbxw7

**DOI:** 10.1186/s12935-015-0253-6

**Published:** 2015-11-26

**Authors:** Jiayu Chen, Haihe Wang, Jing Wang, Shishun Huang, Wei Zhang

**Affiliations:** Department of Laboratory Medicine, School of Medicine, Taizhou University, Taizhou, 318000 Zhejiang China; Department of Pathogenobiology, Daqing Branch of Harbin Medical University, Daqing, 163319 China; Department of Endocrinology of Fifth Hospital of Daqing, Daqing, 163714 China; Department of Pathology, School of Medicine, Zhejiang University, Hangzhou, 310058 China

**Keywords:** STAT1, HCC, Cell cycle arrest, Apoptosis, SMMC7721, HepG2

## Abstract

**Background:**

Aberrant STAT1 signaling is observed in human hepatocellular carcinoma (HCC) and has been associated with the modulation of cell proliferation and survival. However, the role of STAT1 signaling in HCC and its underlying mechanism remain elusive.

**Methods:**

We transiently transfected pcDNA3.1-STAT1 and STAT1 siRNA into SMMC7721 and HepG2 cells. Western blot and qRT-PCR examined the expression of protein and RNA of target genes. Cell viability was assessed using MTT assay, and cell cycle and apoptosis were analyzed by flow cytometry.

**Results:**

We found that STAT1 overexpression increased protein expression of p53 and Fbxw7, and downregulated the expression of cyclin A, cyclin D1, cyclin E, CDK2, Hes-1 and NF-κB p65. These changes led to growth inhibition and induced G0/G1 cell cycle arrest and apoptosis in SMMC7721 and HepG2 cells. Conversely, ablation of STAT1 had the opposite effect on p53, Fbxw7, Hes-1, NF-κB p65, cyclin A, cyclin D1, cyclin E and CDK2, and improved the viability of SMMC7721 and HepG2 cells.

**Conclusions:**

Our data indicate that STAT1 exerts tumor-suppressive effects in hepatocarcinogenesis through induction of G0/G1 cell cycle arrest and apoptosis, and may provide a basis for the design of new therapies for the intervention of HCC in the clinic.

## Background

Hepatocellular carcinoma (HCC) accounts for 80–90 % of liver cancers and is the eighth most commonly occurring cancer in the world [1]. Epidemiological studies have revealed that cirrhosis with hepatitis virus infection is the most predominant risk factor for HCC development [2]. Emerging lines of evidence indicate that aberrant activation of several signaling cascades, including the activator of transcription (Jak/STAT), epidermal growth factor receptor (EGFR), Ras/extracellular signal-regulated kinase (ERK), CDKN1C/P57, phosphoinositol 3-kinase (PI3K)/mammalian target of rapamycin (mTOR) [3], Cyclooxygenase-2/Snail/E-cadherin, NF-κB [4–7] and HGF/cMET pathways [8, 9] contributes to the pathogenesis of HCC. Our previous study has indicated that the mRNA and protein expression of STAT1 were significantly downregulated in the HCC tumor tissues compared to the normal tumor-adjacent tissues [10]. However, the mechanisms underlying the disruption of these critical pathways in the tumorigenesis of HCC are still not fully elucidated.

There is compelling evidence that STAT1 play an important role in promoting apoptotic cell death, and has been supported by the findings that growth inhibiting and pro-apoptotic activities of interferon-gamma (IFN-γ) are largely mediated by STAT1 signaling [11]. It has been observed that IFN-γinduce apoptotic sensitization of cells to various death signals such as TNF-α, Fas, TRAIL [12, 13]. Our previous study pointed out that STAT1 may inhibit HCC growth by regulating the p53-related cell cycling and apoptosis in HepG2 cell [10]. Increasing evidence now suggests that STAT1 signaling also mediates non-apoptotic cell death, a category which includes necrotic and autophagic cell death. The mechanisms of non-apoptotic pathways, which often involves reactive oxygen species (ROS) and a caspase-independent pathway. In addition, STAT1 pathway may play an important role in antiviral defense, inflammation, and injury in liver disease [14]. STAT1 is crucial for the control of hepatitis C virus (HCV) replication although the HCV core protein can selectively degrade STAT1, and subsequently subvert the Jak-STAT kinase [15]. It is possible that STAT1 negatively regulate the growth of HCC. However, how STAT1 regulates the growth of HCC has not been clarified.

In this present study, we tested the impact of induction or knockdown of STAT1 expression on the proliferation, apoptosis and cell cycle of HCC cell line SMMC7721 and HepG2. We have demonstrated that STAT1 upregulation can significantly inhibit the in vitro growth of SMMC7721 and HepG2. Conversely, STAT1 knockdown promoted proliferation in the same cell line. STAT1-induced growth suppression is at least partially due to apoptosis and G0/G1 cell cycle arrest. Consistent with cell cycle arrest, expression levels of cyclin A, cyclin D1, cyclin E, and CDK2 protein all decreased. Upregulation of p53 and Fbxw7 expression and downregulation of NF-κB p65 and Hes-1 were observed, and may be related to STAT1-induced apoptosis. Therefore, Our data suggest that STAT1 may be a negative regulator of the development and progression of human HCC growth through induction of apoptosis and cell cycle arrest.

## Results

### STAT1 overexpression induces apoptosis and cell cycle arrest in SMMC7721 and HepG2 cells

**Fig. 1:**
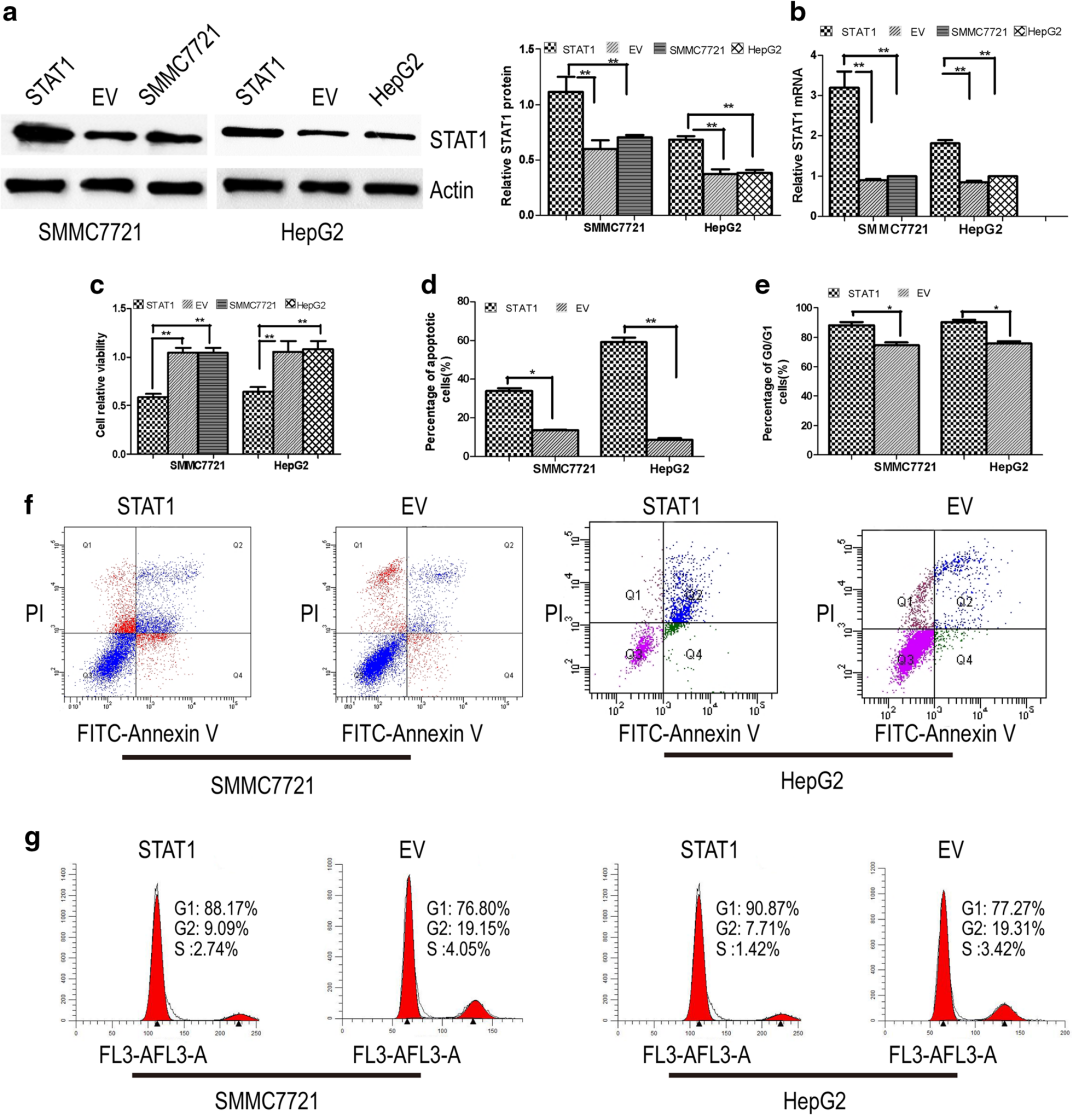
Effects of STAT1 overexpression on SMMC7721 and HepG2 cells growth. **a**, **b** SMMC7721 and HepG2 cells were transfected with an expression vector containing STAT1 and empty vector (EV), or they were left untreated, the levels of STAT1 expression were determined by qRT-PCR and western blot assays; **c** cell proliferation was quantified by MTT assay. STAT1-transfected SMMC7721 and HepG2 cells grew significantly more slowly than EV or untreated cells; **d**, **f** apoptosis was monitored by flow cytometry. The percentage of early apoptotic cells was quantified, and the fraction of apoptotic STAT1-transfected SMMC7721 and HepG2 cells were higher than that of EV cells (P < 0.05); **e**, **g** cell cycle was monitored by flow cytometry. STAT1-transfected SMMC7721 and HepG2 cells showed a higher proportion of cells in G0/G1 phase (88.17 and 90.87 %) compared with control cells (76.80 and 77.27 %) (P < 0.05)

SMMC7721 and HepG2 cells were engineered to transiently express high levels of a recombinant plasmid encoding the STAT1 sequence (pcDNA3.1-STAT1) or control empty vector (EV) pcDNA3.1. The levels of STAT1 expression were determined by qRT-PCR and western blot assays. Compared with the control, the levels of STAT1 proteins in the pcDNA3.1-STAT1-transfected cells showed higher expression than that in the EV and unmanipulated cells (Fig. 1a). Similarly, the levels of STAT1 mRNA in the pcDNA3.1-STAT1-transfected cells were significantly higher than that in the EV and unmanipulated cells (Fig. 1b). The results of the MTT assay (Fig. 1c) indicate that overexpression of STAT1 caused growth inhibition in SMMC7721 and HepG2 cells. The pcDNA3.1-STAT1-transfected cells grew significantly more slowly than that in the EV and unmanipulated cells (P < 0.05). We monitored apoptosis in cultured cells by staining them with Annexin V and Propidium Iodide (PI) for subsequent flow cytometry analysis. We found that apoptosis was more prevalent in pcDNA3.1-STAT1-transfected cells than in EV (P < 0.05) (Fig. 1d, f). Furthermore, flow cytometry analysis was used to measure the cell cycle distribution, as predicted, pcDNA3.1-STAT1-transfected SMMC7721 and HepG2 cells showed a higher proportion of cells in G0/G1 phase (88.17 and 90.87 %) compared with control EV cells (76.80 and 77.27 %) (P < 0.05), indicating that STAT1 significantly inhibited cell cycle progression (Fig. 1e, g), suggesting that STAT1 induces G0/G1 cell cycle arrest and apoptosis in SMMC7721 and HepG2 cells.

### Knockdown of STAT1 expression activates proliferation of SMMC7721 and HepG2 cells

**Fig. 2:**
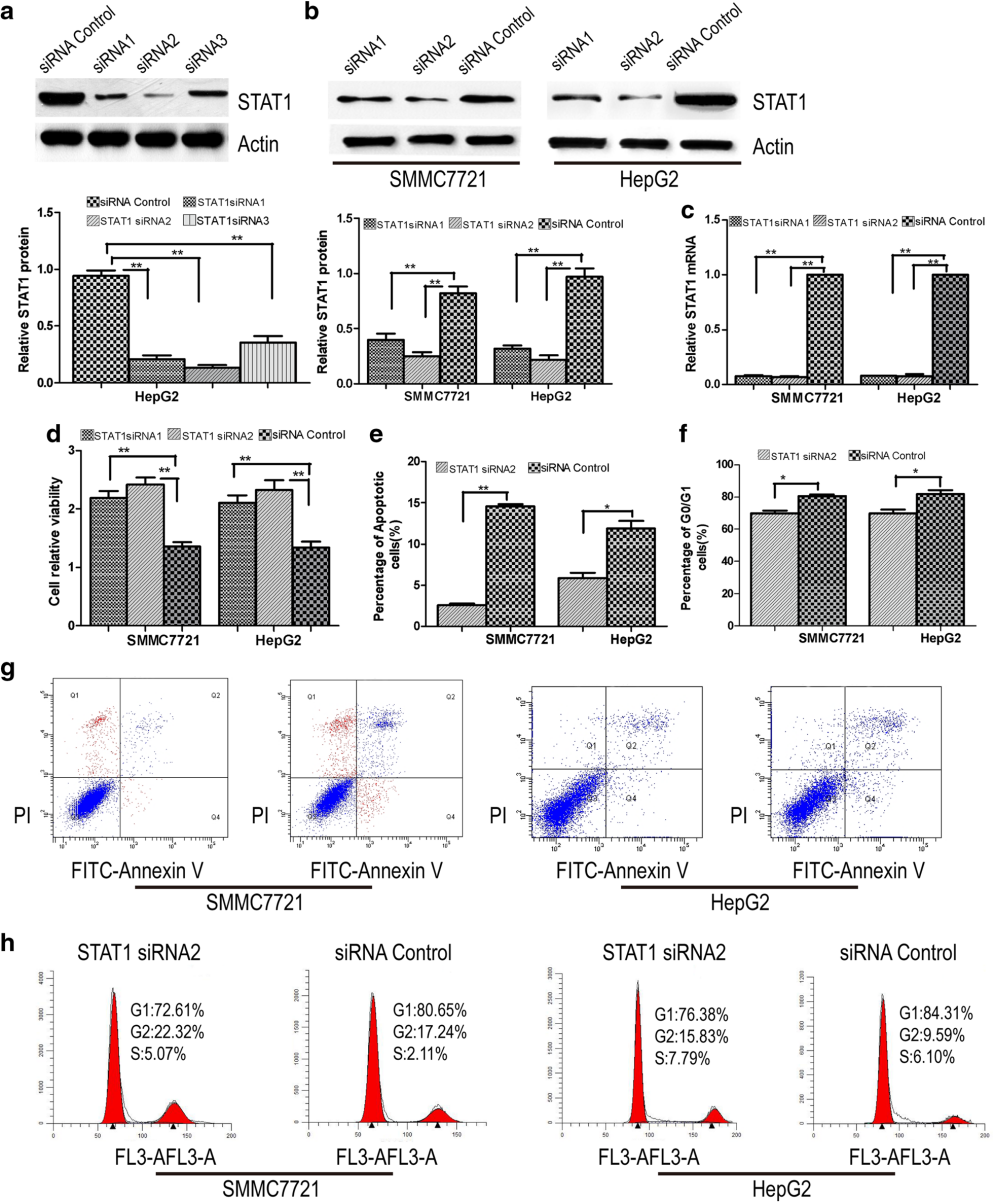
Effects of STAT1 knockdown on SMMC7721 and HepG2 cells growth. **a**, **b**, **c** qRT-PCR and western blot assays were used to determine siRNAs efficiency; **d** cell proliferation was quantified by MTT assay. STAT1 siRNA-transfected SMMC7721 and HepG2 cells grew significantly faster than control siRNA-transfected cells (P < 0.05); **e**, **g** apoptosis was detected by flow cytometry. The percentage of early apoptotic cells was quantified, and the fraction of apoptotic cells was lower in STAT1 siRNA2-transfected cells than in those transfected with control siRNA (P < 0.05); **f**, **h** STAT1 knockdown significantly increased the cell cycle progression. The percentage of G0/G1 phase in STAT1 siRNA2 cells (72.61 and 76.38 %) was lower than that of control siRNA cells (80.65 and 84.31 %) (P < 0.05)

SMMC7721 and HepG2 cells were used to monitor cell survival and growth based on cell cycle and apoptosis after transfection with STAT1 siRNA or control siRNA. To test siRNAs efficiency, STAT1 transcript levels were evaluated by western blot and qRT-PCR assays. We found that all of the tested siRNA effectively reduced the levels of STAT1 expression and transfection with the siRNA1, siRNA2, and siRNA3, resulted in inhibition of STAT1 expression by nearly 75, 86, and 65 % (Fig. 2a, b, c). MTT assay revealed that the ablation of STAT1 improved SMMC7721 and HepG2 cells viability, the STAT1 knockdown cells growing significantly faster than control siRNA-transfected or untreated cells (P < 0.05) (Fig. 2d). Flow cytometry analysis was used to analyze the cell cycle and rate of apoptosis. The result showed that STAT1 knockdown lowered the rate of apoptosis in SMMC7721 and HepG2 cells (P < 0.05, Fig. 2e, g) compared with control siRNA-transfected cells (P < 0.05). STAT1 knockdown significantly increased the cell cycle progression. The percentage of G0/G1 phase in siRNA-transfected SMMC7721 and HepG2 STAT1 cells (72.61 and 76.38 %) was lower than that of control siRNA cells (80.65 and 84.31 %) (Fig. 2f, h).

### STAT1 alters the expression of apoptosis-related proteins in SMMC7721 and HepG2 cells

**Fig. 3:**
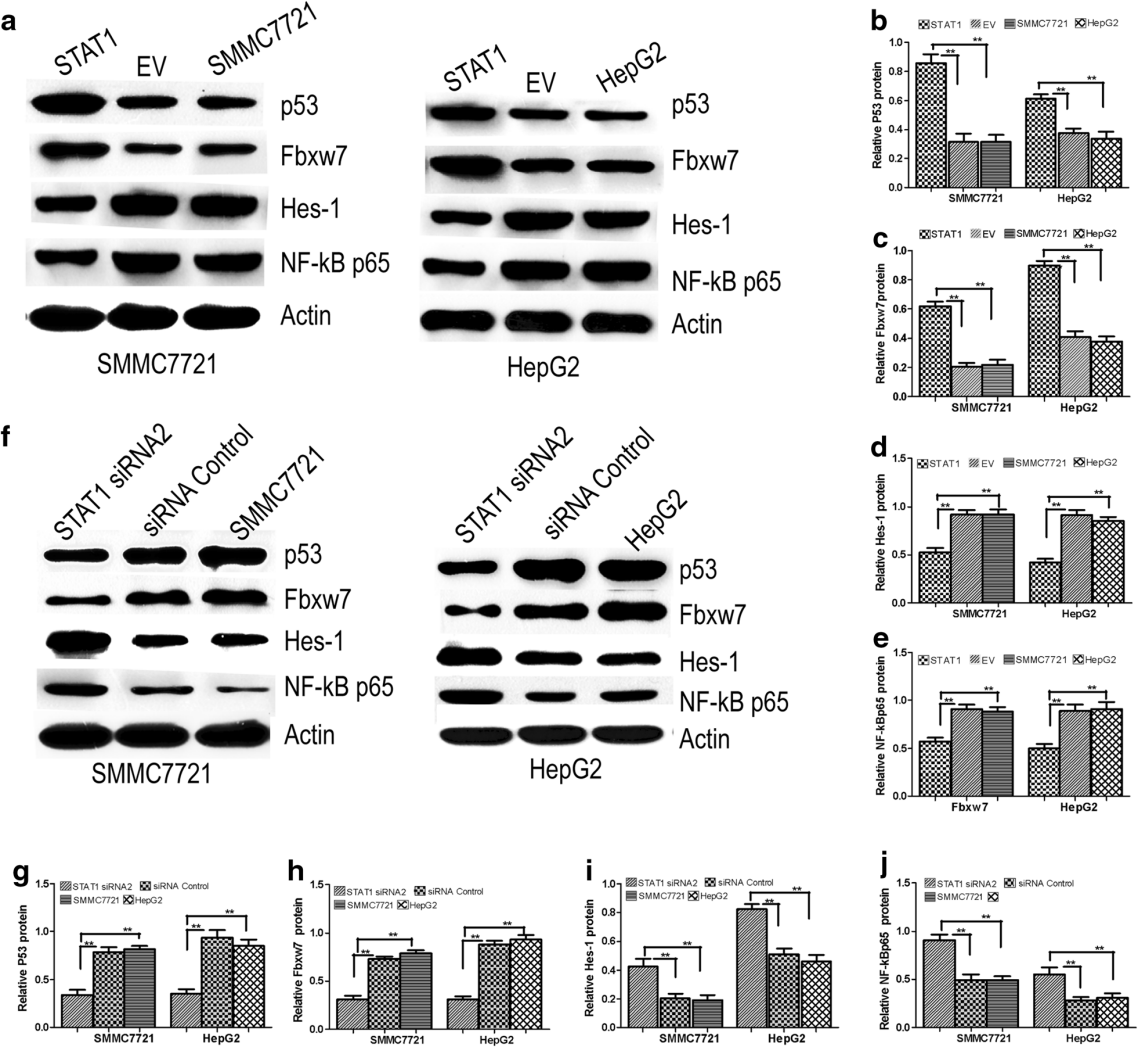
Effect of STAT1 on p53, Fbxw7, Hes-1 and NF-κB p65. **a**, **b**, **c**, **d**, **e** Western blot was used to analyze p53, Fbxw7, Hes-1 and NF-κB p65 protein. Actin served as internal control. p53 and Fbxw7 were significantly increased, Hes-1 and NF-κB p65 were significantly decreased in STAT1-transfected SMMC7721 and HepG2 cells compared to SMMC7721, HepG2 and EV cells (P < 0.05); **f**, **g**, **h**, **i**, **j** showed p53, Fbxw7, Hes-1 and NF-κB p65 protein expression in STAT1 siRNA2, control siRNA, SMMC7721 and HepG2 cells. The protein of p53 and Fbxw7 were significantly decreased, Hes-1and NF-κB p65 were significantly increased in STAT1 siRNA2 cells compared to control siRNA, SMMC7721 and HepG2 cells (P < 0.05)

We analyzed the expression of apoptosis-related proteins p53, Fbxw7, Hes-1, and NF-κB p65 via western blot and qRT-PCR. Western blot analysis showed that transient pcDNA3.1-STAT1 induced a significant increase in p53 and Fbxw7 expression in SMMC7721 and HepG2 cells (P < 0.05, Fig. 3a, b, c), while the expression of Hes-1 and NF-κB p65 were significantly decreased compared with SMMC7721, HepG2 and EV cells, (P < 0.05, Fig. 3a, d, e). We also monitored the mRNA expression of these proteins using qRT-PCR and obtained similar results (not shown). After transfection with STAT1 siRNA2 or control siRNA, Hes-1 and NF-κB p65 expression were increased (Fig. 3f, i, j), whereas p53 and Fbxw7 were down-regulated in STAT1 siRNA2 cells compared with control siRNA-transfected cells (P < 0.05) (Fig. 3f, g, h).

### STAT1 down-regulates the expression of cyclin A, cyclin D1, cyclin E, CDK2 in SMMC7721 and HepG2 cells

**Fig. 4:**
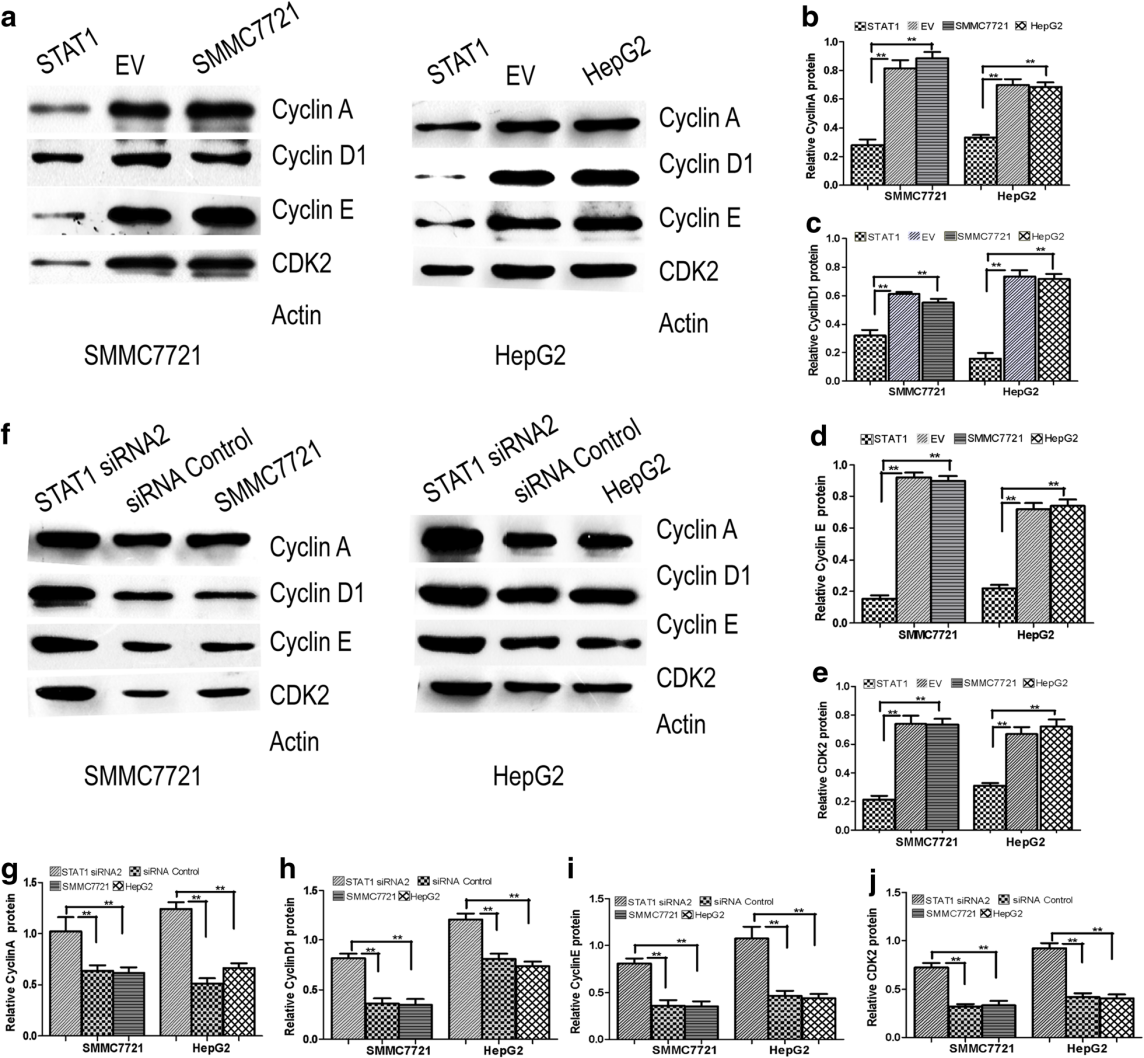
Effect of STAT1 on cyclin A, cyclin D1, cyclin E and CDK2. **a**, **b**, **c**, **d**, **e** Western blot was used to analyze cyclin A, cyclin D1, cyclin E, and CDK2 protein expression. Actin served as internal control. Cyclin A, cyclin D1, cyclin E, and CDK2 expression levels were significantly decreased in STAT1-transfected SMMC7721 and HepG2 cells compared to SMMC7721, HepG2 and EV cells (P < 0.05); **f**, **g**, **h**, **i**, **j** cyclin A, cyclin D1, cyclin E, and CDK2 protein were analyzed by western blot in STAT1 siRNA2, control siRNA, SMMC7721 and HepG2 cells. Cyclin A, cyclin D1, cyclin E and CDK2 expression levels were increased in STAT1 siRNA2 cells when compared with control siRNA, SMMC7721 and HepG2 cells (P < 0.05)

Western blot analysis showed that 48 h after transient transfection with pcDNA3.1-STAT1, cyclin A, cyclin D1, cyclin E and CDK2 expression levels were significantly decreased (P < 0.05, Fig. 4a, b, c, d, e), when compared with SMMC7721, HepG2 and EV cells. In contrast, cyclin A, cyclin D1, cyclin E and CDK2 expression levels were significantly increased 48 h after transient transfection with STAT1 siRNA2 when compared with control siRNA-transfected cells (P < 0.05, Fig. 4f, g, h, i, j). The mRNA expression of these proteins were consistent with those obtained by western blot (not shown).

## Discussion

The signal transducers and activators of transcription (STATs) belong to a family of seven cytoplasmic proteins that function as signal messengers and transcription factors participating in cellular responses to cytokines and growth factors [16, 17]. Among these proteins, the STAT1 plays a critical role in the regulation of diverse biological actions, including antiviral defense, induction of cell death, and growth arrest [18].

In this study, we transiently transfected STAT1 expression vectors and STAT1-specific siRNA into SMMC7721 and HepG2 cells to investigate the function of STAT1 in driving HCC development or progression. We observed that STAT1 overexpression caused growth inhibition, induced G0/G1 cell cycle arrest and promoted apoptosis in SMMC7721 and HepG2 cells, whereas ablation of STAT1 improved viability. The role of the STAT1 cascade in tumor is controversial, and despite strong data indicating that STAT1 downregulation was most prominent in the tumor cells themselves when compared with the surrounding stroma and infiltrating lymphocytes [19, 20]. Studies demonstrated that STAT1 controls anti-tumorigenic effects in part by upregulation of caspases 1, 2, 3, 7, and 8 [21], cyclin-dependent kinase inhibitor 1A (CDKN1A) [22], IFN-regulatory Factor 1 (IRF1)/p53 pathway [23], and down-regulation of B cell CLL/Lymphoma 2 (BCL2) family [24]. In contrast, other groups have found that in certain cellular contexts the STAT1 pathway may mediate tumor cell growth. Overexpression of the IFN/STAT1 pathway is associated with poor prognosis in different types of cancer, and selected against fractionated ionizing radiation (IR) and IFN-resistant phenotype from a radiosensitive parental tumor SCC61 [25–27].

Apoptosis and cell cycle arrest involve complex molecular cascades, and dysfunction of various genes may lead to the onset and progression of apoptosis and cell cycle arrest. We explored the mechanism of STAT1-induced apoptosis and cell cycle arrest by assessing the effect of STAT1 on the expression of apoptosis-related factors (p53, Fbxw7, Hes-1, and NF-κB p65) and cell cycle regulators (cyclin A, cyclin D1, cyclin E, and CDK2) in SMMC7721 and HepG2 cells. It is well recognized that STAT1 promotes cell death through mechanisms that are dependent upon transcriptional activation of genes encoding proteins involved in modulating or promoting cell death, such as caspases, death receptors and ligands, iNOS, and Bcl-xL, as well as those involved in cell cycle arrest, such as p21 WAF1. It is also interesting to note that STAT1 can associate with proteins that are directly or indirectly involved in apoptotic cell death, including TRADD, p53, HATs and HDAC. Previous studies have demonstrated that overexpression of mutant or wild-type p53 can result in apoptotic cell death and that p53 can cause cell cycle arrest by transcriptionally upregulating p21 [28]. Fbxw7 is a key tumor suppressor that regulates cell proliferation in different HCC cell lines. It has been shown that Fbxw7 is directly regulated by p53 [29]. High levels of Fbxw7 might decrease the pool of available cell cycle regulators (cyclin A, cyclin E), triggering arrest in G1 phase and accounting for low proliferation rates. NF-κB signaling has been recognized as the major pathway responsible for cytokine-associated cancer development, inactivation of NF-κB inhibits cell growth and induces intrinsic apoptosis in hepatocellular carcinoma cells [30]. Protein levels and kinase activities of cyclin A, cyclin D1, cyclin E and CDK4 are significantly elevated in HCC, and decreased levels of cyclin D1 have been correlated with growth inhibition and G0-G1 cell cycle arrest [31, 32]. Here, we found a similar effect with STAT1 modulation in HCC cell lines. Our results showed that upon transient expression of active STAT1 in SMMC7721 and HepG2 cells, p53 and Fbxw7 expression levels were increased, whereas cyclin A, cyclin D1, cyclin E, CDK2, Hes-1 and NF-κB p65 were downregulated. STAT1 knockdown by siRNA caused downregulation of p53 and Fbxw7, and upregulation of cyclin A, cyclin D1, cyclin E, CDK2, Hes-1 and NF-κB p65. Taken together, our results suggest that STAT1 possibly induces G0-G1 cell cycle arrest through a mechanism of downregulating cyclin A, cyclin D1, cyclin E, CDK2, and Fbxw7 synergizing with p53 to trigger apoptosis in vitro. To the best of our knowledge, our study provides the first evidence for STAT1-induced G0/G1 cell cycle arrest and apoptosis in SMMC7721 and HepG2 cells.

## Conclusions

In summary, our data indicated that STAT1 may function as a suppressor of HCC cell proliferation, and a regulator of HCC cell apoptosis and cell cycle arrest. Hence, our findings may provide a basis for the design of new therapies for the intervention of HCC in the clinic.

## Methods

### Cell culture and transfections

SMMC7721 and HepG2 cells were cultured in Dulbecco’s modified Eagle’s medium (DMEM) supplemented with 10 % fetal calf serum at 37 °C and 5 % CO_2_. The plasmids of pcDNA3.1 (EV) and pcDNA3.1-STAT1 were obtained from GenePharma. pcDNA3.1 (EV) and pcDNA3.1-STAT1 were transfected into SMMC7721 and HepG2 cells at 70 % confluence using X-tremeGENE HP DNA transfection reagent (Roche, Mannheim, Germany) according to the manufacturer’s protocol. Cells were transfected for 48 h in 6-well plates and then analyzed for flow cytometry, MTT assay, western blot and qRT-PCR.

### RNA interference (RNAi)

The sense and antisense sequences of the STAT1-specifc and control siRNAs were synthesized by GenePharma, Shanghai, China and are present in Table 1. SMMC7721 and HepG2 cells (2 × 10^5^/well) were cultured in 6-well plates at 70 % confluence, and transfected in duplicate with 1.3 µg each type of STAT1-specific or control siRNA using X-tremeGENE siRNA transfection reagent (Roche, Mannheim, Germany) according to the manufacturer’s instructions. Cells were transfected for 48 h in 6-well plates and then analyzed for flow cytometry, MTT assay, western blot and qRT-PCR.

**Table 1:** Primer for PCR amplification and siRNAs sequences

Sequencing primers	5′–3′
p53-F primer	CAGATCCCTTAGTTTTGGGTGC
p53-R primer	GCCTGGAGAGACCTAGACCA
Fbxw7-F primer	AAAGAGTTGTTAGCGGTTCTCG
Fbxw7-R primer	CCACATGGATACCATCAAACTG
HES-1-F primer	CCAAAGACAGCATCTGAGCA
HES-1-R primer	TCAGCTGGCTCAGACTTTCA
NF-κB p65-F primer	TCTGCAACTGAAACGCAAGC
NF-κB p65-R primer	CTCCACAGCTTCTCTTACCTT
CDK2-F primer	CGGATCTTTCGGACTCTGGG
CDK2-R primer	GAGAGGGTGAGCCGATTAGG
CyclinA-F primer	CAGAGGCCGAAGACGAGAC
CyclinA-R primer	TCAGCTGGCTTCTTCTGAGC
CyclinD1-F primer	CTGGCCATGAACTACCTGGA
CyclinD1-R primer	GTCACACTTGATCACTCTCC
CyclinE-F primer	GTTATAAGGGAGACGGGGAG
CyclinE-R primer	TGCTCTGCTTCTTACCGCTC
RITA-F primer	GGGTGGAGAAGGCTAACAGAA
RITA-R primer	CATCACAGTAAGACGGGGTGT
GAPDH-F primer	AGGTGAAGGTCGGAGTCAAC
GAPDH-R primer	CGCTCCTGGAAGATGGTGAT
siRNA1:STAT1_homo-575 sense	GCUGGAUGAUCAAUAUAGUTT
siRNA1:STAT1_homo-575 antisense	ACUAUAUUGAUCAUCCAGCTT
siRNA2:STAT1_homo-647 sense	GCGUAAUCUUCAGGAUAAUTT
siRNA2:STAT1_homo-647 antisense	AUUAUCCUGAAGAUUACGCTT
siRNA3:STAT1_homo-1601 sense	GCACCUGCAAUUGAAAGAATT
siRNA3:STAT1_homo-1601 antisense	UUCUUUCAAUUGCAGGUGCTT
Control-siRNA-sense	UUCUCCGAACGUGUCACGUTT
Control-siRNA-antisense	ACGUGACACGUUCGGAGAATT

### Cell cycle determination

After transfection with the corresponding STAT1 vectors or siRNAs for 48 h, the cells were harvested and washed twice with cold PBS, then fixed in 75 % alcohol for 2 h at 4 °C. After washing in cold PBS three times, cells were resuspended in 1 mL of PBS solution with 40 μg of propidium iodide (Sigma) and 100 μg of RNase A (Sigma) for 30 min at 37 °C and analyzed with the BD Accuri C6 system (Becton–Dickinson, USA). The distribution of cells in different phases of the cell cycle was calculated using the Modifit LT software.

### Apoptosis analysis

After transfection for 48 h, the cells were harvested and washed three times with cold PBS. Cells were then resuspended in the staining buffer and stained using the Annexin V-FITC Apoptosis Assay Kit (Bestbio, Shanghai, China) according to the manufacturer’s instructions. The stained cells were analyzed by flow cytometry (BD FACSAria, R&D, USA). Annexin V-positive and propidium iodide-negative cells were counted as apoptotic cells.

### MTT assay

Cell viability was assessed using the MTT colorimetric assay (R&D, USA). SMMC7721 and HepG2 cells were seeded in 96-well plates at a density of 1 × 10^4^ cells per well in 100 μL complete medium. After transfection with the corresponding plasmid vector or siRNA, 10 μL MTT solution (5 mg/mL) was added, and cells were incubated for an additional 4 h. Subsequently, 100 µL of MTT solubilization buffer was added to the wells. Following a 10-min mixing period, the absorbance was analyzed at 570 nm using a microplate reader. The background absorbance at 690 nm was subtracted from the 570 nm measurement. Each experiment was performed in triplicate, and the mean value was calculated.

### Quantitative Real-time reverse transcription-polymerase chain reaction (qRT-PCR)

Total RNA was isolated from cultured cells using Trizol (Invitrogen, Carlsbad, California, USA) according to the manufacturer’s instructions. RNA concentration and purity were determined from absorbance measured at both 260 and 280 nm. RNA (1 μg) was reverse transcribed using the PrimeScript^®^ First Strand cDNA Synthesis Kit (Takara, Dalian, China) and random oligodeoxynucleotide primers. PCR amplification was performed in 20 μL reactions containing cDNA generated from 2 ng of the original RNA template, 400 nmol/L of each gene-specific forward and reverse primer, 10 μL of 2 × SYBR^®^ Premix Ex Taq™ II (Takara, Dalian, China), 0.4 μL of ROX reference dye, and 6.0 μL of dH_2_O. Amplified signals were detected using the ABI PRISM 7300 Real-Time PCR system (ABI, USA). The annealing temperature was optimized using the temperature gradient program that is part of the ABI PRISM software. Experiments were performed in duplicate. The relative target mRNA levels were determined using the 2^ΔΔCt^ method. The primer sequences used are summarized in Table 1.

### Western blot analysis

Protein concentrations were determined using the BCA Protein Assay (Pierce, USA) according to the manufacturer’s instructions. Samples containing equal amounts of protein (40 μg) were resolved by 10 % SDS-PAGE and transferred to nitrocellulose membranes. After blocking for 2 h at room temperature with TBS-T (0.1 M Tris, 0.9 % NaCl, and 0.05 % Tween-20 at pH 7.5) containing 5 % skim milk, and probed at 4 °C overnight with rabbit anti-STAT1 (1:2000, Bioworld Technology), rabbit anti-p53 (1:400), rabbit anti-Fbxw7 (1:400), rabbit anti-cyclin A (1:400), rabbit anti-cyclin D1 (1:400), rabbit anti-cyclin E (1:400), rabbit anti-CDK2 (1:400) (Beijing Biosynthesis Biotechnology Co, Beijing, China), rabbit anti-NF-κB p65 (1:2000), rabbit anti-Hes-1 (1:2000) (Cell Signaling Technology Inc., Boston, USA), and mouse anti-actin (1:8000). Proteins were detected by exposing the blots to X-ray film (Kodak).

### Statistical analysis

SPSS version 17.0 software was used for all statistical analyses. All of the results are expressed as the mean ± SD. Statistical analysis was performed using standard two-way ANOVA for repeated measurements, and the Χ^2^ test was used to analyze the flow cytometry data. P values less than 0.05 were considered statistically significant.
